# Scale-up of the Physical Activity 4 Everyone (PA4E1) intervention in secondary schools: 24-month implementation and cost outcomes from a cluster randomised controlled trial

**DOI:** 10.1186/s12966-021-01206-8

**Published:** 2021-10-23

**Authors:** Rachel Sutherland, Elizabeth Campbell, Matthew McLaughlin, Nicole Nathan, Luke Wolfenden, David R. Lubans, Philip J. Morgan, Karen Gillham, Chris Oldmeadow, Andrew Searles, Penny Reeves, Mandy Williams, Nicole Evans, Andrew Bailey, James Boyer, Christophe Lecathelinais, Lynda Davies, Tom McKenzie, Katie Robertson, John Wiggers

**Affiliations:** 1 Hunter New England Population Health, Locked Bag 10, Wallsend, NSW 2287 Australia; 2 School of Medicine and Public Health, University of Newcastle, Callaghan, NSW 2308 Australia; 3 Hunter Medical Research Institute, New Lambton Heights, NSW 2305 Australia; 4 Priority Research Centre for Health Behaviour, School of Medicine and Public Health, University of Newcastle, Callaghan, NSW 2308 Australia; 5 South Western Sydney Local Health District, Locked Mail Bag, 7279, BC1871, Liverpool, NSW Australia; 6 Central Coast Local Health District, 4-6 Watt Street, Gosford, NSW 2250 Australia; 7 Mid North Coast Local Health District, P.O. Box 126, Port Macquarie, NSW Australia; 8 New South Wales Department of Education, School Sports Unit, Level 3, 1 Oxford Street, Darlinghurst, NSW 2010 Australia

**Keywords:** Physical activity, Adolescents, School, Randomised controlled trial, Implementation, Multi-component, Scale-up, Cost-effectiveness, Fidelity, Reach

## Abstract

**Background:**

Physical Activity 4 Everyone (PA4E1) is an evidence-based program effective at increasing adolescent physical activity (PA) and improving weight status. This study aimed to determine a) the effectiveness of an adapted implementation intervention to scale-up PA4E1 at 24-month follow-up, b) fidelity and reach, and c) the cost and cost-effectiveness of the implementation support intervention.

**Methods:**

A cluster randomised controlled trial using a type III hybrid implementation-effectiveness design in 49 lower socio-economic secondary schools, randomised to a program (*n* = 24) or control group (*n* = 25). An adapted implementation intervention consisting of seven strategies was developed to support schools to implement PA4E1 over 24-months. The primary outcome was the proportion of schools implementing at least four of the 7 PA practices, assessed via computer assisted telephone interviews (CATI) with Head Physical Education Teachers. Secondary outcomes included the mean number of PA practices implemented, fidelity and reach, cost and cost-effectiveness. Logistic regression models assessed program effects.

**Results:**

At baseline, no schools implemented four of the 7 PA practices. At 24-months, significantly more schools in the program group (16/23, 69.6%) implemented at least four of the 7 PA practices than the control group (0/25, 0%) (*p* < 0.001). At 24-months, program schools were implementing an average of 3.6 more practices than control schools (4.1 (1.7) vs. 0.5 (0.8), respectively) (*P* < 0.001). Fidelity and reach of the implementation intervention were high (> 75%). The total cost of the program was $415,112 AUD (2018) ($17,296 per school; $117.30 per student).

**Conclusions:**

The adapted implementation intervention provides policy makers and researchers with an effective and potentially cost-effective model for scaling-up the delivery of PA4E1 in secondary schools. Further assessment of sustainability is warranted.

**Trial registration:**

Australian New Zealand Clinical Trials Registry ACTRN12617000681358 prospectively registered 12th May 2017.

**Supplementary Information:**

The online version contains supplementary material available at 10.1186/s12966-021-01206-8.

## Introduction

Physical inactivity has been well documented as a risk factor for chronic disease and a significant contributor to the burden of disease in Australia and internationally [1, 2]. Despite the undisputed benefits of physical activity (PA), 81% of adolescents aged 11–17 years globally do not meet the current World Health Organization (WHO) recommendations of 60 min of moderate-to-vigorous PA (MVPA) per day [2]. As a result, widespread implementation of school-based PA programs at a population level have been recommended [3].

Adolescence is a key life stage, where investments could yield triple benefits – e.g. benefits today, into adulthood and into the next generation [4]. Despite the acknowledged benefits of a universal prevention approach, few school-based PA programs have been implemented at scale [5], particularly targeting adolescents. For example, recent reviews of PA programs implemented at scale found no programs targeting adolescents in the secondary school setting [6]. This is perhaps unsurprising given very few school-based PA programs targeting adolescents have demonstrated effective outcomes [7, 8]. Of the limited number of programs effective at increasing adolescent PA [7, 8], those tested under ideal research conditions may not be amenable for implementation at scale in real-world contexts, as they require expertise and resources not readily available within schools [7, 8]. Consequently, policy makers and practitioners select programs that require substantial adaptations for implementation at scale [9, 10], diluting their effectiveness [6]. Carefully planned adaptations to the implementation approach to support scale-up may overcome such challenges [9].

Physical Activity 4 Everyone (PA4E1) is an evidence-based PA program targeting secondary schools located in economically disadvantaged areas [11]. PA4E1 consists of a multi-strategy implementation support intervention (herein referred to as the ‘implementation intervention’) that aims to support schools to implement seven evidence-based PA practices, which have been shown to increase adolescent PA and improve weight status in a previous efficacy trial conducted from 2012 to 2014 [12–15]. The efficacy trial of PA4E1 cost AUD $329,952 over 24-months ($394 per student; $65,990 per school), equating to $56 ($35–$147) per additional minute of moderate to vigorous intensity PA [13]. For decision makers, outcomes relating to cost and economic analyses are an important consideration for scale-up [16]. To support the scale-up of PA4E1, the implementation intervention was adapted [11] to explicitly incorporate theoretically derived implementation support strategies. A 24-month randomised controlled trial of this adapted PA4E1 program was initiated in 2017. Results at 12-months (mid-program) demonstrated a significant increase in school PA practice implementation in the program schools compared to control schools, with 67% (16/24) of schools in the program arm implementing at least four of the seven school PA promoting practices compared to 4% (1/25) of schools in the control arm (OR = 33 [4–1556], *p* < 0.001) [17].

To address the need to scale-up and evaluate PA programs targeting adolescents, this paper extends on the previously reported 12-month outcomes [17] by evaluating the longer-term impacts at the completion of the 24-month implementation intervention. This paper reports the effectiveness, fidelity and reach, cost and cost-effectiveness of the implementation intervention on the implementation of school PA practices.

## Methods

### Study design and setting

**Fig. 1:**
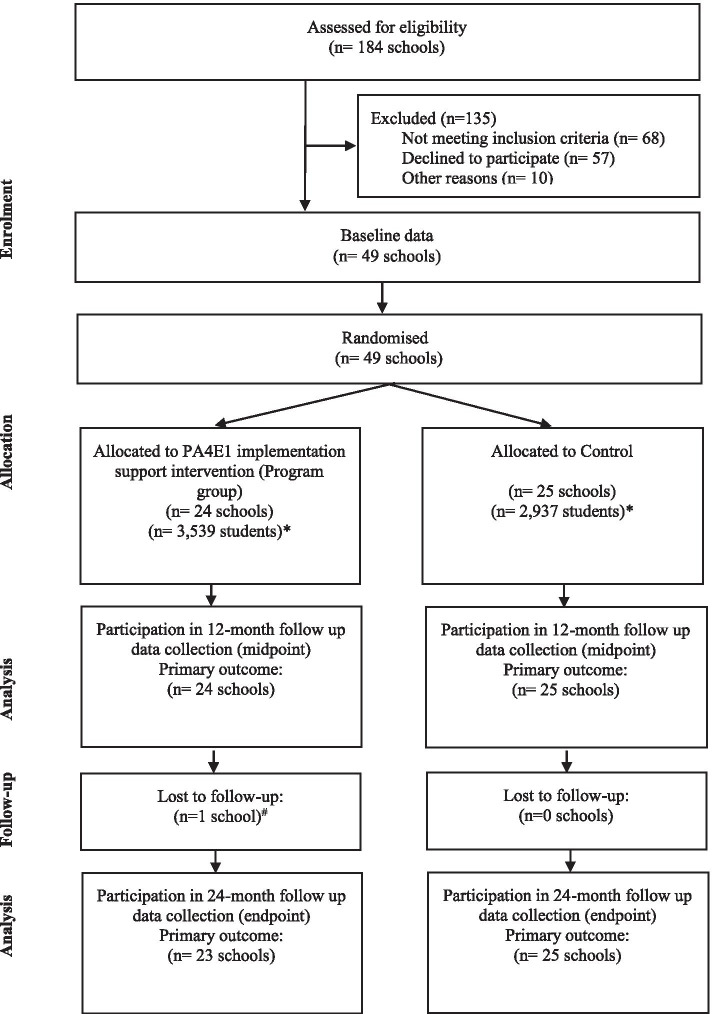
Consort Flow Diagram describing progress to 24 months (final follow-up). *Grade 7 enrolments [19]. # of Head PE Teacher

Details of the trial methods have been previously reported [11, 18]. Briefly, this trial employed a type III hybrid implementation-effectiveness design with 49 secondary schools located in New South Wales (NSW), Australia (Fig. 1). Secondary schools in NSW cater for 11–18 year olds (Grade 7–12). There are 509 secondary schools in NSW [19]. The three types of schools, Government, Catholic and Independent, represent 71, 18 and 11% of all schools, respectively [19, 20]. This paper reports the school-level implementation of PA practices (primary outcome), fidelity and reach of the implementation support strategies, and program costs and cost-effectiveness at completion of the 24-month implementation intervention (secondary outcomes). In line with the published protocols [11, 18], separate papers will report the secondary trial outcomes, such as PA outcomes and a comprehensive process evaluation [11, 18].

The trial adheres to Consolidated Standards of Reporting Trials (CONSORT) Statement (Supplementary File 1), the Standards for Reporting Implementation Studies (StaRI) Statement and checklist (Supplementary File 2) and the Template for Intervention Description and Replication (TIDieR) checklist (Supplementary File 3).

### Sample, participants and recruitment

Schools were recruited from four NSW Local Health Districts (LHD); Mid-North Coast, South-Western Sydney, Central Coast and Hunter New England. Schools were eligible if they were: a) not involved in the original PA4E1 trial; b) Government or Catholic schools; c) enrolled students from grades 7–9; d) not a specialist academic/ sporting/ performing arts/ agriculture/ boarding school; e) located in areas classified as being disadvantaged by the SEIFA Index of Relative Socio-economic Disadvantage (suburb in lower 50% of NSW) [20]; and f) not participating in other major whole school PA trials or initiatives.

Eligible schools were stratified by LHD and school sector (Government or Catholic). A letter outlining the study was emailed to the school principal requesting the information be shared with Head Physical Education (PE) teachers. Secondary schools in NSW have a Head PE teacher who leads the Health and PE teachers. A PE trained project officer employed by the research team then contacted the principal or Head PE teacher to invite school participation. Face-to-face meetings and additional telephone meetings were offered to further outline the study requirements, confirm eligibility and gain written consent. In consenting schools, all PE teachers were emailed a study information sheet. At baseline, 12-month and 24-months, Head PE teachers were emailed an invitation to participate in a computer assisted telephone interview (CATI). Consent was implied by completing the CATI.

### Randomisation and blinding

Stratified block randomisation was used to allocate consenting schools to one of two groups in a 1:1 ratio. Separate random block sequences of sizes two and four were used within each of eight strata. The blocks were created through SAS version 9.3. Population of the blocks involved using a random number generator in Microsoft Excel to randomise the order of the schools, prior to pasting into their respective stratum block. This was conducted by a statistician not involved in recruitment and blinded during the randomisation phase. Principals were notified by research staff of their school’s allocation to either the program (intervention) or usual care control following baseline collection of school practice measures (Head PE teacher CATI). Schools were not blinded to group allocation, however interviewers conducting the CATI were not informed of group allocation nor was the statistician undertaking the randomisation and analysis.

### Evidence-based PA program (PA4E1) – program group

The program involved two components:

1. Physical activity practices

The program protocol paper [11], process evaluation protocol paper [18] and 12-month midpoint outcomes paper [17] provide a detailed description of 7 PA4E1 school PA practices. A detailed description of adaptations to the original efficacy trial made for scale-up is reported separately [18]. The 7 PA practices were (i) quality PE Lessons; (ii) student PA plans; (iii) enhanced school sport program; (iv) recess/ lunchtime PA; (v) school PA policy or procedure; (vi) links with community PA providers; (vii) communicating PA messages to parents. The PA practices are further outlined in Supplementary File 4.

2) Implementation intervention

Table 1 outlines the implementation intervention, consisting of seven overarching implementation strategies, within which there were 23 sub-strategies. The Behaviour Change Wheel and the Theoretical Domains Framework were used to develop the implementation intervention [11]. Implementation support was delivered over eight school terms (two school years) as described in Table 1.

**Table 1:** Overview of the implementation intervention delivered over 24 months (8 school terms), and the fidelity (provided), reach (uptake) and cost ($AUD) of each strategy

***Implementation support strategies (n = 7) and sub-strategies (n = 23) (implemented over 8 school terms) of the implementation intervention***	***Fidelity (provided)*** ***(n/N schools)***			***Reach (uptake)*** ***(n/N schools)***			***Mean cost per school over 24 months***
	0–12 months	12–24 months	0–24 months	0–12 months	12–24 months	0–24 months	($AUD)
**1. Executive and leadership support**							108.50
**1.1:** PA4E1 Partnership agreement signed by school executive.	N/A	N/A	N/A	24/24	24/24	24/24	
**1.2:** New or existing school committee formed to oversee program.	N/A	N/A	N/A	15/24	18/24	11/24	
**1.3:** The School committee is inclusive of in-School Champion and school executive to oversee the program.	N/A	N/A	N/A	10/24	11/24	7/24	
**1.4:** Committee met at least once per term.	N/A	N/A	N/A	10/24	13/24	8/24	
**2. Embedded school staff: in-School Champion**							14,000.00
**2.1:** An existing school PE teacher is allocated the role of in-School Champion by schools to support implementation.	N/A	N/A	N/A	24/24	23/24	23/24	
**2.2:** The position was funded by the NSW Department of Health, half day per week (equivalent to $350AUD a fortnight).	24/24	24/24	24/24	24/24	24/24	24/24	
**3. External implementation support**^**b**^							723.50
**3.1:** Health Promotion Support Officer (ideally a trained PE teacher) appointed to support schools with the program.	24/24	24/24	24/24	24/24	24/24	24/24	
**3.2:** Health Promotion Support Officer was co-located within the relevant local health district.	17/24	24/24	17/24	17/24	24/24	17/24	
**3.3**^bc^**:** Weekly contact was made with in-School Champion via phone, email and/or face-to-face site visits for the first 12 months. In the second 12 months, phone, email and/or face-to-face contact was made according to the following high dose/low dose protocol matched to school practice uptake in the preceding term (e.g. term 5 informs term 6) ○ Schools implementing four or less practices received 2 face-to-face contact points, as well as 6 emails or phone calls in the term. ○ Schools implementing five or more practices received 1 face-to-face contact point and seven emails or phone calls in the term.	N/A	N/A	N/A	N/A	N/A	N/A	
**3.4**^b^**:** Support Officer and in-School Champion have a face-to-face contact at least once a term for the first 12 months. In the second 12 months, the Support Officer and in-School Champion have face-to-face site visits according to the following high dose/low dose protocol matched to school practice uptake in the preceding term (e.g. term 5 informs term 6) ○ Schools implementing four or less practices received 2 face-to-face contact points per term ○ Schools implementing five or more practices 1 face-to-face contact point per term	24/24	24/24	24/24	18/24	8/24	5/24	
**4. Teacher professional learning**							588.29
**4.1:** In-School Champion training −1-day of face to face training was hosted per year by PA4E1 implementation. Accommodation, meals and transport costs were covered by the NSW Department of Health.	24/24	24/24	24/24	24/24	22/24	22/24	
**4.2:** Quality PE training for all PE teachers – 6 × 10-min online training videos followed by knowledge check short quizzes focused on the SAAFE principles were delivered via a password protected program website.	24/24	21/24	21/24	12/24	11/24	7/24	
**4.3**^a^**:** Enhanced school sport training – in-School Champions and other teachers involved in delivering the program could attend an existing 1 day face-to-face Resistance Training for Teens workshop offered by the NSW Department of Education (School Sport Unit), or equivalent training run by PA4E1 implementation team (not accredited). Course costs to be paid by project for in-School Champion, but not for other teachers.	24/24	24/24	24/24	23/24	23/24	23/24	
**4.4**^a^**:** School physical activity policy training – in-School Champion offered existing online training run by the NSW Department of Education School Sport Unit (Government schools only, *n* = 19) [33].	19/19	19/19	19/19	4/19	4/19	4/19	
**5. Resources**							775.21
**5.1**^a^**:** Printed posters outlining Quality PE principles (SAAFE Principles [2]) to be displayed in PE department delivered to in-School Champions.	24/24	24/24	24/24	24/24	24/24	24/24	
**5.2**^a^**:** A $100AUD physical activity equipment voucher was provided to support the delivery of recess and lunchtime physical activity.	24/24	24/24	24/24	24/24	24/24	24/24	
**5.3**^a^**:** Equipment provided to support the delivery of recess and lunchtime physical activity enhanced schools sport program (5 Gymsticks/school).	24/24	24/24	24/24	22/24	22/24	22/24	
**5.4:** Electronic resources housed on the program website (PA4E1 online) included: - overview of program presentation (Microsoft PowerPoint presentation) - project milestones to be achieved each term (over 4 terms) - online quality PE training (SAAFE Principle videos (6 videos – one overview and one per Principle) and worksheet, peer observation materials) - student personal physical activity plan templates - recess and lunch resources - policy templates - examples of community physical activity providers - tips and frequently asked questions	24/24	24/24	24/24	24/24	24/24	24/24	
**6. Provision of prompts and reminders**							0.00
**6.1:** Weekly contact was made with in-School Champion via phone, email and/or face-to-face site visits for the first 12 months. In the second 12 months, contact was made by the Support Officer to in-School Champions via phone, email and/or face-to-face site visits according to the following high dose/low dose protocol matched to school practice uptake in the preceding term (e.g. term 5 informs term 6) ○ Schools implementing four or less practices received 2 face-to-face contact points, as well as 6 emails or phone calls in the term. ○ Schools implementing five or more practices 1 face-to-face contact point and seven emails or phone calls in the term.	16/24	22/24	16/24	16/24	22/24	16/24	
**6.2:** Automated messages were sent each term via the program website to in-School Champions to prompt completion of teacher professional learning and online termly performance monitoring and feedback surveys.	24/24	24/24	24/24	24/24	24/24	24/24	
**7. Implementation performance monitoring and feedback**							16.25
**7.1**: In-School Champion completes all termly surveys via the program website (PA4E1 Online).	24/24	21/24	21/24	24/24	21/24	21/24	
**7.2**: A feedback report is automatically generated and sent to in-School Champions via email	24/24	24/24	24/24	24/24	24/24	24/24	
**7.3**: A feedback report is automatically generated and sent to school Principals via email.	24/24	24/24	24/24	6/24	7/24	6/24	
**Additional Centralized Resources**							1075.00
Cost to develop and maintain the PA4E1 online portal used by all schools in the program group to automate the delivery of the implementation strategies and house resources electronically.							
**Total**	**95.6%**	**98.1%**	**94.4%**	**81.5%**	**82.5%**	**75.5%**	**$17,296.33**

Total fidelity and reach scores are percentage across all schools. Only sub-strategies with available data were included. Sub-totals for fidelity and reach percentages were calculated within each implementation strategy first, then sub-totals were averaged from all seven strategies to produce a final fidelity and reach percentage

N/A Excluded as there was no available data

Supplementary File 6 outlines the data sources and criteria used to determine fidelity and reach for each implementation support strategy (sub-strategies 1.1–7.3) at both 0–12 months and 12–24 months

Supplementary File 7 outlines a more detailed results table for the implementation support strategy cost per year and mean cost per school

^a^Only offered in the first 12 months, scores from 0 to 12 months carried over to 12–24 months

^b^ Support offered by the external Support Officer was standardized across all school between baseline and 12-months. Between 12 and 24 months, support offered by the external support officer was tailored depending on the progress of the school towards desired milestones, as described in each sub-strategy

^c^Data was collected, but sub-strategy 3.3 is a combination of sub-strategy 3.4 and 6.1, the data was not counted twice and is hence listed as not applicable

### Control group

Schools randomly allocated to the control group received no contact from the research team other than to participate in the study data collection at baseline, 12 and 24-months. These schools continued with their usual practices for delivering PE lessons, school sport and other PA programs and practices.

### Data collection procedures and measures

Baseline data were collected August–October 2017, at 12-months from September–December 2018 and at 24-months from September–December 2019 following completion of the 24-month program. Schools were offered half a day of teacher relief ($175AUD) at each data collection point to reimburse for time.

#### Primary trial outcome

The primary outcome was the proportion of schools in each group implementing any four of the 7 PA practices at 24-months. Our prior efficacy trial demonstrated a significant effect on objectively measured student MVPA [12] when the implementation of four of the 7 PA practices was achieved by program schools. Measures of school practices were undertaken via CATI with Head PE teachers, administered by trained interviewers. The CATI items were pilot tested and forwarded to all participants via email before data collection. The questions relating to the implementation of the school practices within the current school year (2017 at baseline, 2018 at 12-months, 2019 at 24-months) are available in Supplementary File 5. For program schools, if Head PE teachers were not in the role of in-School Champion, they were asked to discuss (prior to the CATI) their schools involvement and uptake of the PA4E1 program with their PA4E1 in-School Champion. Telephone interviews (including CATI) regarding PA practice implementation have successfully been used in large studies with validity and reliability and achieve high response rates [21, 22].

#### Secondary outcomes

##### Mean number of school practices

Mean number of school PA practices implemented and whether or not schools implemented each of the seven practices were assessed via CATI as described for the primary outcome.

##### Fidelity and reach of implementation support strategies

To determine the proportion of schools that were provided (fidelity) and took up (reach) each of the seven implementation strategies (Table 1), we used: project records kept by External Support Officers, website usage data, termly implementation audit and feedback surveys completed by PA4E1 in-School Champions and Head PE teacher CATI. See Supplementary File 6 for an outline of the criteria for fidelity and reach.

##### Economic outcomes

Economic outcomes included the mean incremental cost per school and mean incremental cost per student to implement the program, mean cost per implementation support strategy and the incremental cost-effectiveness ratio (ICER). Incremental cost is the difference in the mean cost per student between study groups and the incremental effect is the difference in the primary trial outcome. The incremental cost–effectiveness ratio (ICER) represents the additional cost to achieve a percent change in the proportion of schools implementing at least four of the seven school PA practices (reflective of the primary trial outcome) [11]. Resource use was captured through a bespoke cost-capture tool. Economic outcomes are described in detail in Supplementary File 7.

##### School characteristics/ head PE teacher characteristics

Information regarding school sector, postcode, size (total enrolments), Indigenous enrolments, and students who speak a language other than English (LOTE) was assessed via publicly available data [23]. Additional information including number of PE teachers and full time equivalent PE positions at the school; language groups most commonly represented (when the school has more than 10% of students from LOTE households), sex of Head PE teacher, PE training, years of teaching experience, how long they’ve taught PE at their current school, and grades they teach in the current year were obtained through the Head PE teacher CATI.

### Sample size

Using consent rates obtained from previous trials conducted in schools by the research team (65–70% consent) [14], a sample of 76 schools (38 per arm) was estimated to provide 80% power to detect an absolute increase of ~ 35% between groups in the proportion of schools implementing at least four of the seven practices at 24-months. Without prior data on baseline levels of school PA practices, this calculation made the assumption that 40% of schools in the control arm could achieve this target at follow-up.

### Statistical analyses

All analyses were conducted using SAS, version 9.3, from February to April 2020. Characteristics of schools participating in the trial and those refusing participation were compared using Chi-square analyses and these results are reported in the 12-month midpoint outcomes paper, see Sutherland et al. [17]. School characteristics were summarised for program and control schools [17]. Analysis followed intention to treat principles, where schools were analysed according to their randomised treatment allocation. Given the low prevalence of practices at baseline, the number of schools per group, and almost no implementation by control schools at follow-up, the generalised linear regression models planned were not undertaken. Instead, dichotomous outcomes (implementing at least four of the seven practices (primary outcome) (yes, no) and each of the seven practices (secondary)) at 24-months were analysed using exact logistic regression models adjusting for baseline and for the stratification variables (LHD, school sector). For consistency in analysis approach, a linear regression model was used to assess differences between groups at 24-months on the continuous secondary outcome variable (number of PA practices), adjusting for baseline and the stratification variables. Significance levels for the analyses were set at *p* < 0.025 as per the trial protocol [11] to allow for program effects at 12 or 24-months.

Descriptive statistics were used to summarise the fidelity and reach of the implementation intervention over 0–12 months (previously published) [17], 12–24 months and 0–24 months (see Supplementary File 6 for further details). Per protocol analyses were undertaken for the primary outcome and one secondary outcome as described in Supplementary File 6. Economic analysis methods are described in Supplementary File 7.

### Ethics approval

The trial was prospectively registered (ACTRN12617000681358) and approved by the Hunter New England Research Ethics Committee (Ref No. 11/03/16/4.05), University of Newcastle (Ref No. H-2011-0210), NSW Department of Education (SERAP 2011111), Maitland Newcastle Catholic School Diocese, Broken Bay Catholic School Diocese, Lismore Catholic School Diocese, Armidale Catholic School Diocese, and the Aboriginal Health and Medical Research Council.

## Results

### Sample characteristics

Figure 1 outlines the flow of schools and participants through the study. There were 184 secondary schools assessed for eligibility of which 78 schools were ineligible due to not meeting the inclusion criteria. Of the remaining 106 schools, 49 consented and 57 declined (Fig. 1, 46% consent rate). The reasons for declining [17] primarily related to lack of executive support, capacity to nominate an in-School Champion and concerns regarding allocation to the control group. There were no significant differences in the characteristics of schools declining to participate compared to consenting schools based on sector, LHD, size, percentage enrolment of Indigenous students or students speaking a language other than English (LOTE), except schools that declined were more likely to have 10% or more students of LOTE background (24/57 42% of refusers, 8/49 16% of consenting, *p* = < 0.05). The characteristics of participating schools at baseline are provided in Table 2.

**Table 2:** Characteristics of program and control schools at baseline and respondents at 24 months

***Characteristic***	***Description***	***Program (n = 24)*** ***n***			***Control (n = 25)*** ***n***		
***School Characteristics***							
*Secondary school in eligible Local Health Districts*	All eligible Local Health Districts	24			25		
	- Central Coast	2			3		
	- Hunter New England	13			12		
	- Mid North Coast	5			6		
	- South Western Sydney	4			4		
*School sector*	Government	19			21		
	Catholic	5			4		
*Remoteness*	Major Cities	11			13		
	Inner Regional	9			10		
	Outer Regional/Remote	4			2		
*School socioeconomic status* *(SEIFA)*	Decile 1 (Most disadvantaged in State)	8			9		
	Decile 2	7			3		
	Decile 3	2			5		
	Decile 4	6			6		
	Decile 5	1			2		
*School size (total enrolments)*	Small (< 400)	2			2		
	Medium (400–800)	8			17		
	Large (> 800)	14			6		
*Grade 7 size*^a^	Small (< 65)	3			3		
	Medium (65–135)	7			14		
	Large (> 135)	14			8		
	**Total Students**	**3539**			**2937**		
*Female enrolments*	< 30%	0			1		
	30–40%	0			0		
	41–50%	11			15		
	51–60%	6			5		
	100% (female only schools)	2			0		
*Indigenous enrolments*	< 10%	12			10		
	> = 10%	12			15		
	- 10–24%	8			14		
	- 25–49%	4			1		
*Language background other than English*	< 10%	19			22		
	> = 10%	5			3		
	- 10–24%	1			0		
	- 25–49%	0			0		
	- 50–74%	1			2		
	- > = 75%	3			1		
*PDHPE faculty size*	1–4 FTE (3–12 staff)	5			6		
	5–8 FTE (4–13 staff)	16			17		
	9–13 FTE (9–15 staff)	3			2		
*Respondent (Head PE teacher or delegate)*		*Baseline*	*12 months*	*24 months*	*Baseline*	*12 months*	*24 months*
	**Gender**						
	- Male	16	14	12	16	15	15
	- Female	8	10	11	9	10	10
	**PDHPE trained**						
	- Yes	24	24	23	25	25	25
	**Years of teaching experience**						
	- < 1	0	1	0	0	0	0
	- 1 to 5	3	1	2	0	0	2
	- 6 to 10	6	7	7	4	7	5
	- 11 to 15	1	4	3	6	7	8
	- 16 to 20	5	3	5	5	4	4
	- > 21	9	8	6	10	7	6
	**Years teaching PDHPE at current school**						
	- New in that year	2	1	3	2	1	5
	- < 3	0	3	2	2	3	2
	- 3 to 5	4	3	3	3	3	4
	- > 5	18	17	15	18	18	14

PA4E1 is a whole-school program for students in all grades. For a detailed description of the seven program practices (i.e. evidence-based intervention), see Supplementary File 4

*Abbreviations*: *PDHPE* Personal development, health and physical education, *PE* Physical Education, *SEIFA* Index of relative Socio-economic disadvantage via school suburb

^a^“Grade 7 size” included in the table because two of the curriculum physical activity practices targeted Grade 7 students as the key target group. The student level evaluation was also conducted in student in Grade 7

### Primary outcome: implementation of PA practices

**Fig. 2:**
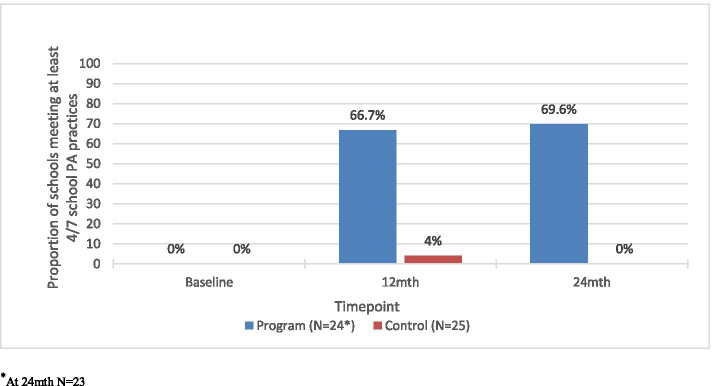
Proportion of secondary schools meeting primary outcome at baseline, 12-months and 24-months (primary outcome). ^*^At 24mth *N* = 23

Table 3 presents the school PA practice results, (including the previously published results at 12-months [17]) and the 24-month results. Two schools had missing data for one practice at baseline, and one school did not complete the 24-month follow-up CATI. For the primary outcome, at baseline no schools were implementing four of the seven school PA practices in the current school year. At 24-month follow-up, significantly more schools were implementing four of the seven school PA practices in the program group (16/23, 69.6%) than the control group (0/25, 0%) (*p* < 0.001). These results remained consistent even when the school missing 24-month data was included in the analysis as not meeting any practice. Figure 2 shows the proportion of schools meeting the primary outcome, implementation of four or more practices at baseline, 12 and 24-months, by group. Supplementary File 8 shows the number of program group schools meeting each practices at neither, 12-months only, 24-months only, or both 12- and 24-month follow-ups.

**Table 3:** School implementation of PA practices at baseline, 12 and 24 month follow-up

School practice implementation in current school year	Program Group Baseline *N* = 24% (n)	Program Group 12 months *N* = 24% (n)	Program Group 24 months *N* = 23% (n)	Control Group Baseline *N* = 25^a^ % (n)	Control Group 12 months *N* = 25% (n)	Control Group 24 months *N* = 25% (n)	***P*** value 0–12 months^**b**^	OR (95% CI) 0–24 months	***P*** value 0–24 months
**Primary outcome** – 4 or more PA practices	0.0 (0)	66.7 (16)	69.6 (16)	0.0 (0)	4.0 (1)	0.0 (0)	***< 0.001***	***42.5 (8.6-∞)***	***< 0.001***
**Secondary outcomes**									
Mean number of PA practices (SD)	0.5 (0.8)	3.9 (1.5)	4.1 (1.7)	0.5 (0.7)	0.7 (1.0)	0.5 (0.8)	***< 0.001***	***β = 3.6 (2.8–4.4)***	***< 0.001***
Meeting each PA practice									
1. Quality PE lessons *Incorporating desirable elements*^*c*^	8.3 (2)	58.3 (14)	60.9 (14)	4.0 (1)	4.0 (1)	8.0 (2)	***< 0.001***	***13.9 (2.6–143.7)***	***< 0.001***
	8.3 (2)	54.2 (13)	60.9 (14)	4.0 (1)	4.0 (1)	8.0 (2)	***< 0.001***	***13.9 (2.6–143.7)***	***< 0.001***
2. Student PA plans *Incorporating desirable elements* ^*c*^	8.3 (2)	87.5 (21)	82.6 (19)	0.0 (0)	8.0 (2)	8.0 (2)	***< 0.001***	***47.0 (5.9–2186.9)***	***< 0.001***
	0.0 (0)	8.3 (2)	26.1 (6)	0.0 (0)	4.0 (1)	4.0 (1)	*1.00*	***6.9 (0.8–333.4)***	*0.10*
3. Enhanced school sport program	0.0 (0)	83.3 (20)	69.6 (16)	4.0 (1)	4.0 (1)	4.0 (1)	***< 0.001***	***31.9 (4.3–1404.2)***	***< 0.001***
4. Recess/lunchtime PA *Incorporating desirable elements* ^*c*^	8.3 (2)	50.0 (12)	60.9 (14)	24.0 (6)	28.0 (7)	16.0 (4)	0.059	***13.5 (2.3–157.9)***	***< 0.001***
	*0.0 (0)*	*33.3 (8)*	*56.5 (13)*	*8.0 (2)*	*8.0 (2)*	*4.0 (1)*	0.027	***35.7 (6.8-∞)***	***< 0.001***
5. PA policy or procedure	0.0 (0)	29.2 (7)	39.1 (9)	4.0 (1)	4.0 (1)	0.0 (0)	0.044	***22.0 (4.0-∞)***	***< 0.001***
6. Links with community PA providers *Incorporating desirable elements* ^*c*^	4.1 (1)	0.0 (0)	8.7 (2)	0.0 (0)	4.0 (1)	0.0 (0)	1.0	3.1 (0.4-***∞)***	0.39
	*4.1 (1)*	*0.0 (0)*	*4.4 (1)*	*0.0 (0)*	*4.0 (1)*	*0.0 (0)*	1.0	*1.3 (0.1-∞)*	0.86
7. Communicating PA messages to parents	20.8 (5)	83.3 (20)	87.0 (20)	^a^16.0 (4)	20.0 (5)	16.0 (4)	***< 0.001***	***53.4 (6.3–2626.1)***	***< 0.001***
**Per protocol** ^**d**^- Program schools median reach or above Primary outcome – 4 or more PA practices (n/N)									
0–12 months	0.0 (0/13)	76.9 (10/13)		0.0 (0/23)	4.0 (1/25)		**< 0.001**		
0-24mths	0.0 (0/15)		76.9 (10/13)	0.0 (0/23)		0.0 (0/25)			**< 0.001**
Secondary outcome - Mean number PA practices (SD)									
0–12 months	0.4 (0.8)	4.2 (1.2)		0.5 (0.7)	0.7 (1.0)		**< 0.001**		
0-24mths	0.5 (0.7)		4.2 (1.6)	0.5 (0.7)		0.5 (0.8)			**< 0.001**

^a^ There were two schools with missing parent practice data in the control group at baseline. These schools were included in the baseline analysis for the primary outcome as ‘not met’ they both had two practices and an additional practice would not have enabled them to meet the four practice criteria

^b^ as reported in 12 month paper [17]

^c^ Supplementary File 4 outlines the details of each physical activity practice, including desired elements

^d^Supplementary File 6 outlines the methods for the per-protocol analyses

### Secondary outcomes

#### Mean number of school practices

**Fig. 3:**
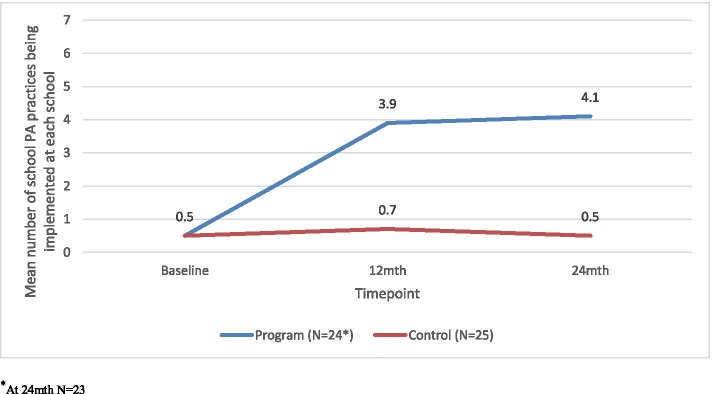
Mean number of practices implemented by program and control group schools at baseline, 12 and 24 months (secondary outcome). ^*^At 24mth *N* = 23

At 24-months, program schools were implementing a mean of 4.1 (1.7 SD) practices and control schools a mean of 0.5 (0.8 SD) practices, resulting in a difference favouring the program group of 3.6 practices more than schools in the control group (< 0.001). For six of the seven practices, schools in the program group were significantly more likely to implement them than the control group (see Table 3). The remaining school PA practice, links with community PA providers, was not significantly different between groups at 24-month follow-up (OR 3.1, 0.4-∞; *p* = 0.39). Fig. 3 shows the mean number of practices being implemented per school at baseline, 12 and 24-months, by group.

#### Per protocol analyses

Per-protocol results are reported in Table 3 and are consistent with the main analyses. The per-protocol program group (*n* = 13) was defined as up-taking (reach) at or above the median reach score for the implementation support strategies. At 24-month follow-up, significantly more per-protocol program schools had implemented four of the seven practices (10/13, 76.9%) than control group schools (0/25, 0.0%) (*p* < 0.001). After adjusting for baseline differences, the per-protocol program group was implementing a mean of 3.7 more practices than the control group at 24 months (*p* < 0.001).

#### Fidelity and reach of the implementation support strategies

Table 1 shows the overall mean fidelity score over 0–24 months (implementation support provided) across all schools was 94.4% (range 86.1–100.0%). Reach (uptake of the implementation support) was also high over 0–24 months at 75.5% (range 64.3–88.1%).

#### Cost and cost-effectiveness

Table 1 outlines the cost of each implementation support strategy per school. The total program cost over 24-months was $415,112 (95%UI $410,245 - $420,851), resulting in an incremental cost per school to receive the implementation intervention over 24-months being $17,296 (95%UI$17,094 - $17,535). Based on 3539 students enrolled in the target year 7, the mean incremental cost per student over 24-months was calculated to be $117.30. The incremental cost-effectiveness ratio was calculated to be $25,944 (95%UI $20,512 - $38,259) per percent increase in the proportion of school implementing at least four of the seven school PA practices.

Supplementary File 7 outlines the total cost and mean cost per school of the implementation intervention from baseline to 12-months, between 12 to 24-months and the overall costs of the implementation intervention across the 24-month program.

## Discussion

### Main findings

This implementation trial provides strong evidence, that with theoretically designed implementation support intended to address identified barriers, schools can overcome commonly reported implementation failure which has plagued many school-based PA trials [7]. At a cost of AUD$17,296 per school, the PA4E1 implementation intervention was effective at increasing secondary schools’ implementation of evidence-based school PA practices at 24-months. More than two thirds (69.6%) of schools in the program group were implementing at least four of the seven PA practices at the desired standard, averaging implementation of 4.1 of the recommended seven practices, whilst control schools implemented a mean of just 0.5 practices and no schools achieving implementation of at least four of the seven practices. Our exploratory per protocol analysis indicated that where uptake of the implementation support was greater, school implementation of practices were even higher, with 76.9% of per-protocol program schools implementing at least four of seven school-based PA practices. Our previous efficacy trial has demonstrated that this level of PA practice implementation within a school translates to greater daily MVPA and slower weight gain in adolescent students [12, 14, 24].

### Findings in context

Whilst there are very few implementation trials of school-based PA programs in secondary schools to enable direct comparisons, a systematic review evaluating the effect of the limited number of school-based PA programs indicates that magnitude of the effect size, measured at the individual level, generally halves when programs are scaled up [6]. Given this, the magnitude of our findings, even though measured at a school level, appears large compared to systematic review findings and studies monitoring the implementation of school-based practices in the real world [5, 6]. For example, the unadjusted median effect size reported in a Cochrane systematic review of school-based implementation trials was just 19% (range 8.5 to 66.6%) [5]. Our study appears consistent with one implementation study undertaken within elementary schools utilising similar strategies to increase schools’ implementation of PA policies at scale, which resulted in an effect size of 40% difference between intervention and control group [25]. Both interventions utilised implementation theory to overcome identified school implementation barriers resulting in common implementation support strategies. These included harnessing executive support, appointing in-School champions, external support officers and the provision of equipment and resources [25].

We found that the first 12-months of the implementation intervention resulted in a sharp increase in school PA practice implementation. This impact appeared to be maintained, but not largely extended between 12 to 24-months (Fig. 3). This is similar to effects seen in the *Live Life Well @ School (LLW@S)* program, a comprehensive obesity prevention program in Australian primary schools. Consisting of ten school nutrition and PA practices, following ongoing implementation support from local health services, the proportion of schools implementing the desirable practices increased from 39.5% in year 2 to 71.4% in year 3 but remained at 72% after 5 years of implementation support. Therefore, intensive implementation support over an extended period may not be necessary to embed evidence-based strategies in a school setting. Instead, future research could examine if a low-intensity support model (at a lower cost) for sustainability may be more appropriate following an intensive initial implementation period.

### Varying PA practice implementation

At an individual practice level, our implementation intervention resulted in significantly higher implementation of six of the seven school PA practices in the program group in comparison to the control schools. Formal links with community sport organisations being the only school PA practice that appeared to have very limited implementation by schools, even after 2 years of support. The challenge with formally working in partnership with community sports providers appears to be shared by other schools, and not unique to this trial [26]. Nonetheless, systematic reviews have demonstrated that school-based programs that include a community component result in greater impacts on student PA levels [27]. As a result, a greater emphasis on determining the school-level barriers and support required to overcome these is warranted. Interestingly, between the 12 and 24-months of the intervention, the implementation of four of the school PA practices (quality PE, recess and lunchtime PA, school PA policy and communicating with parents) increased, whilst the implementation of two school PA practices decreased in the same period (enhanced school sport, and slightly for student PA plans). The implementation of two curriculum based strategies decreased, which may demonstrate they are more challenging for schools to achieve. This is perhaps because schools were required to implement the curriculum strategies across the whole year group of students in order to achieve the practice. Additionally, staff turnover of PE teachers in some schools presented particular challenges.

### Economic evaluation

In order to achieve population health gains, models of implementation need to be effective in changing practice in the real world, but also cost-effective to be implemented at scale or by segments of the community that would benefit most [28]. This 24-month program cost AUD$17,296 per school or AUD$117 per student. Unsurprisingly, there is a paucity of data regarding economic evaluations of implementation support interventions in general [28], but specifically limited empirical data evaluating the cost-effectiveness of school-based studies. In a systematic review of public health focused implementation studies, only one of the 14 included studies focused on economic evaluation [28], this being the cost-effectiveness evaluation of our previous PA4E1 school-based PA efficacy trial [13]. In comparison to our previous efficacy trial [13], which was deemed to be potentially cost-effective at a cost of AUD$65,990 per school, or AUD$394 per student, or AUD$56 per 1 min increase in MVPA, our implementation intervention (which was adapted from the previous efficacy trial for implementation at scale) is considerably less cost per school (74% less) and per student (70% less).

### Comparisons to the pre-scale efficacy trial

The primary outcome of this scale-up trial was the implementation of four or more school PA practices [11], reported here. A secondary outcome is students mean daily MVPA assessed via wrist-worn accelerometer, which will be reported separately in line with methods reported in our trial protocol [11]. In the efficacy trial, the primary outcome was students waist-worn accelerometer measured MVPA, which had significant effects at 12 [12] and 24-month [14] follow-up. In the efficacy trial, implementation of school PA practices was measured via in-school consultant records (different to the current trial which uses Head PE teacher CATI). Although the measures differed, the original trial found a significant effect on student PA with the implementation of 4 of 7 practices [12, 14]. The efficacy trial also found that by 24-months, four of five schools were able to implement all school PA practices. By contrast, only one school in the current trial implemented all practices. It appears that practice implementation in the original trial was therefore higher than the current scale-up trial.

Our previous model of implementation relied primarily on a full time external change agent supporting five schools 1 day per week [14], whilst the current implementation intervention model utilizes a lower cost model, combining a website, an in-School Champion (an existing PE teacher funded 0.5 days per week) and support from an External Support Officer (ideally with PE teacher training) [13]. Given the high level of implementation achieved by schools, but also the relatively high cost of the implementation support, further opportunities to optimize the implementation model [9] should be explored by considering opportunities for further adaptations to reduce the absolute costs, and potentially improving cost-effectiveness. For example, further scale-up of the program could seek to evaluate if schools require a funded in-School Champion position or if this could be allocated within existing school resources. Similarly, if external implementation support could be provided via alternate modes of delivery, other than face-to-face support, further reducing the implementation costs. Evaluating the merit of optimised implementation models could occur via non-inferiority designs, embedded within further scale-up attempts, similar to other novel school-based PA scale-up interventions [25]. A similar process of continual optimization has been applied to a school nutrition program resulting in the development of a more cost-effective model of implementation support and achieving a halving of the ICER through an optimization process [10, 29]. A comprehensive process evaluation will be published separately and will assist decision makers in adapting the program for further scale-up [18].

### Strengths and limitations

The primary outcome of this implementation trial relied on self-report via a Head PE teacher telephone interviews, which may be subject to social desirability. This is a limitation shared by other implementation trials [30], however such methods have also shown to be valid and reliable [21, 22]. Further, it is possible that increased implementation of school PA practices does not guarantee that increased activity is delivered or that all students participate, despite our previous trial which demonstrated an effect on student MVPA and weight status [12, 14, 24]. Finally, the 24-month practice data was collected immediately following the program delivery, as such it remains unknown if schools are able to sustain practice implementation, or further maintenance support would be required [25]. Importantly, even with these limitations, this was a large cluster randomised controlled trial that utilised implementation strategies and behaviour change techniques that were explicitly selected to address known barriers and mapped against a robust theoretical framework, evaluating both implementation and cost-effectiveness. This trial is one of the first implementation trials targeting school-based PA practices in secondary schools globally, therefore addressing a much needed research gap and providing critical evidence regarding future implementation of health programs and practices within secondary schools. Additional process evaluation data will be published elsewhere [18], to facilitate the interpretation of the trial outcomes [17]. Further, this trial was implemented over a 24-month period, which is consistent with systematic reviews of school-based programs suggesting that those of greater than 12-month duration were more likely to be effective. Lastly, it provides one of very few cost-effectiveness evaluations of an implementation support intervention in a real world setting. Taken together, the efficacy, implementation, process and cost evidence from the previous efficacy trial [12–15] and this scale-up trial [11, 17, 18] suggests this implementation intervention may be suitable for further scale-up at a population level (e.g. state-wide dissemination) [16, 31, 32].

## Conclusion

This study provides robust evidence of an implementation intervention that is effective and potentially cost-effective to support the scale-up of evidence-based PA practices in secondary schools, a setting and target group in critical need of empirical evidence. To date, secondary school-based PA interventions have been limited by poor implementation, limiting their effectiveness to impact on student physical activity outcomes. Policy makers and researchers looking to support secondary schools to implement efficacious school PA practices should consider the use of a theoretically designed implementation intervention targeting known barriers to implementation.

## Supplementary Information

**Additional file 1 **: **Supplementary File 1**. CONSORT Checklist.

**Additional file 2 **: **Supplementary File 2**. StaRI Checklist.

**Additional file 3 **: **Supplementary File 3.** TIDieR Checklist.

**Additional file 4 **: **Supplementary File 4**. Overview of the evidence-based PA4E1 program (physical activity practices) including standards required of program schools (essential elements) and additional desirable elements.

**Additional file 5 **: **Supplementary File 5**. Overview of the interview questions assessing school physical activity practice implementation.

**Additional file 6 **: **Supplementary File 6**. Fidelity, reach and per-protocol analyses.

**Additional file 7 **: **Supplementary File 7**. Economic evaluation.

**Additional file 8 **: **Supplementary File 8**. Practice implementation for each practice.

## Data Availability

All study materials are available from the research team upon request to the corresponding author. All data are stored securely as per ethical requirements. All participants were issued a unique identification number following consent for confidentiality. The final trial dataset is stored securely and accessed only by the study statistician. The results of this trial are being disseminated via publications in peer reviewed journals, conference presentations, reports to schools and relevant health and education departments.

## Abbreviations

PA: Physical Activity
PA4E1: Physical Activity 4 Everyone
MVPA: Moderate-to-vigorous intensity physical activity
LOTE: Language other than English
NSW: New South Wales
PE: Physical Education
PDHPE: Personal development, health and physical education
CATI: Computer assisted telephone interviews
