# Supplementary material for: Scale-up of the Physical Activity 4 Everyone (PA4E1) intervention in secondary schools: 24-month implementation and cost outcomes from a cluster randomised controlled trial
Source: Int J Behav Nutr Phys Act. 2021 Oct 23;18:137. doi: 10.1186/s12966-021-01206-8 (PMC8542325; doi:10.1186/s12966-021-01206-8)
Supplement: Supplementary file 4 — Additional file 4 : Supplementary File 4. Overview of the evidence-based PA4E1 program (physical activity practices) including standards required of program schools (essential elements) and additional desirable elements. [file 12966_2021_1206_MOESM4_ESM.docx]

## Supplementary File 4

**Supplementary File 4, Table 1:** Overview of the evidence-based PA4E1 program (physical activity practices) including standards required of program schools (essential elements) and additional desirable elements ([16](#_ENREF_16))

| ***Physical activity practices by Health Promoting Schools domain*** |
| --- |
| *Curriculum, teaching and learning* |
| 1. **Quality Physical Education (PE) lessons:**  - PE department used documented principles or guidelines for teachers to maximise PE quality, active learning time and student engagement in PE lessons (Program schools used the SAAFE principles- Supportive, Autonomous, Active, Fair, Enjoyable) ([44](#_ENREF_44)). - Each PE teacher also participated in peer observation of a practical PE lesson, at least once a year*. - *Desirable – peer observation feedback is against the department’s quality PE principles.* |
| 1. **Student physical activity (PA) plans:**  - All Grade 7 students developed a personal PA plan which included  1. personal goals to improve or maintain activity or fitness 2. actions and timelines to achieve goals and 3. progress monitoring  - Goals reviewed once within year - *Desirable – students in Grades 7-10 develop a personal PA plan*** |
| 1. **Enhanced school sport program:**  - The school delivered a short (10-12 weeks) structured PA Program (i.e., Resistance Training for Teens ([45](#_ENREF_45))) designed to target resistance training and improve adolescents’ fitness and provide them with knowledge, motivation and skills to engage in a range of lifelong physical activities. - The program should be delivered to all students in at least one Grade between 7 and 10 (Program schools delivered the Resistance Training for Teens program to all of Grade 7 ([46](#_ENREF_46)). |
| *Ethos and environment* |
| 1. **Recess/ lunchtime physical activity:**  - Supervised recess and/or lunchtime PA sessions offered to all students in Grades 7-10 at least 3 days per week. - PA equipment freely available to students at least three days per week at recess and/or lunch. - *Desirable - at least one organised recess or lunch activity per week targeting girls. Sessions promoted to students at least once per term.*      1. **School PA Policy or Procedure:**  - School developed a policy which included:  1. Provision of at least 150 minutes/week of MVPA during school time for all students in Grade 7-10; 2. Supportive practices to enhance all students’ PA (at least 3 of practices 1-4, 6-7 in this table)*** |
| *Partnerships and services* |
| 1. **Links with community physical activity providers:**  - School has at least three links that went beyond promotion of the provider (e.g. in newsletters) to involve an agreement, connection, partnership (e.g. out of hours sessions on school facilities, presentation by providers at school). - Links were designed to support ‘outside of school time’ activity. - Links were communicated to students and families at least once per term****. - *Desirable - at least one of the community links made were to promote free or low-cost options in the community.*  1. **Communicating physical activity messages to all parents**  - All parents of students in Grades 7-10 received PA messages that were designed to increase parent knowledge, attitudes and support towards PA, at least once per term. - These excluded messages only about school events e.g. carnivals, or school sports timetables or results, or promotion (advertisements for) community PA providers).**** |

*Program schools were asked to aim for peer observations once a semester

** Program schools asked to set plans for Grade 7 at 12 and 24-months

*** Program schools asked to include practices 1-4, 6 and 7 in their policy.

**** Program schools asked to use multiple modes to promote community links and to communicate PA information to parents (e.g. newsletters, parent app, parent information evening)
