# Supplementary material for: Scale-up of the Physical Activity 4 Everyone (PA4E1) intervention in secondary schools: 24-month implementation and cost outcomes from a cluster randomised controlled trial
Source: Int J Behav Nutr Phys Act. 2021 Oct 23;18:137. doi: 10.1186/s12966-021-01206-8 (PMC8542325; doi:10.1186/s12966-021-01206-8)
Supplement: Supplementary file 5 — Additional file 5 : Supplementary File 5. Overview of the interview questions assessing school physical activity practice implementation. [file 12966_2021_1206_MOESM5_ESM.docx]

## Supplementary File 5

### Supplementary File 5, Table 1: Overview of the computer assisted telephone interview (CATI) questions assessing school physical activity practice implementation

| **Physical activity practices within the current school year** | **Response Scale** | **Criteria for implementation**  **(schools to meet all criteria in this column for the practice)**  ***(additional criteria for desirable practice elements – encouraged in program schools)*** |
| --- | --- | --- |
| **1. Quality PE lessons** |  |  |
| Does your department use an agreed set of principles or guidelines for PE teachers to maximize PE quality, active learning time and student engagement in PE lessons? | 1.Yes written document  2.Yes but not as written document  3. No | Yes written document |
| Please describe or name the principles.* | Open | SAAFE principles or at least two of the component principles (Supportive, Autonomous, Active, Fair, Enjoyable) - when reviewed by two PE teachers within the research organization. Disagreements resolved through discussion. |
| Does your PE department do peer observation?** (defined as using a structural observation framework to provide feedback on quality practical PE lessons – could be linked to professional development or accreditation – could include observation by a PE teacher or expert external or internal to the school). | 1. Yes  2. No  3. Don’t know | Yes |
| How often does your school do peer observation? | 1.Once per term  2.Once per semester  3.Once per year  4.Don’t know | Once year or more |
| *Do the peer observations provide feedback against the quality PE principles you mentioned?* | 1.Yes  2. No  3.Don’t know | *Yes (for desirable)* |
| **2. Student physical activity plans** |  |  |
| Does your PE department have a process for all students in Grades 7, 8, 9 or 10 to write a personal physical activity plan? (defined as including the following i) include personal goals to improve or maintain regular activity or fitness; ii) student to state specific actions and timelines to achieve their goals; and iii) evaluate their progress and review and revise their goals at least once within the year). | 1. Yes  2. No | Yes |
| Which Grades? | 1. 7  2. 8  3. 9  4.10  5.Don’t know | Grade 7  *Grades 7 - 10 (for desirable)*  *(Note- criteria retained at Grade 7 for outcome assessment although Program schools encouraged to implement plans in Grades 7-10 in second year of project)* |
| **3. Enhanced school sport program:** |  |  |
| Is an enhanced school sport program scheduled in your school program or timetabled as compulsory for all students within Grade 7, 8, 9 or 10? (defined as: short duration (10-12 weeks) physical activity program that can be run during school sport time or another spare period; aims to influence student’s motivation for lifelong activity and their activity levels; includes an information component to address physical activity recommendations, benefits and strategies; and teacher directed physical activity sessions). | 1. Yes  2. No  3. Don’t know | Yes |
| Name the program/s.  Provide a document showing an outline of the program (if the program is not Resistance Training for Teens) | Open  Document requested via email on completion of interview | Resistance training for Teens, or another program rated as meeting definition when reviewed by two PE teachers within the research organization based on program name/description in open question and for programs that both rated as query, either program outline (if provided) or searches for named program (if outline not provided). Disagreement resolved through discussion. Programs rated as queries by both teachers after viewing available information were rated as not meeting criteria. |
| Which Grades compulsory for? (for each program nominated) |  | Any Grade 7-10 for at least one program meeting criteria |
| **4. Recess/ lunchtime physical activity** |  |  |
| On average, how many days per week does your school offer planned and supervised physical activities by a teacher or staff member, to all students at recess and/or lunch? | 1. Never  2. <1 day per week  3. 1 day per week  4. 2 days per week  5. 3 days per week  6. 4 days per week  7. 5 days per week | 3 or more days per week |
| Is sporting equipment feely available to all students during recess and/or lunchtime? | 1. Never  2. <1 day per week  3. 1 day per week  4. 2 days per week  5. 3 days per week  6. 4 days per week  7. 5 days per week | 3 or more days per week |
| *How often do you have planned and supervised physical activities at recess and/or lunch that are designed to specifically target girls?* | 1. Never  2. <1 day per week  3. 1 day per week  4. 2 days per week  5. 3 days per week  6. 4 days per week  7. 5 days per week | *One of more days per week (for desirable)* |
| *How often are recess and lunch time activities promoted to all students, for example through assembly announcements, morning roll call, school newsletters, SRC/Sports captains, over a PA system?* | 1. never  2. 1-3 times year  3. once term  4. more than once per term | *Once term of more (for desirable)* |
| **5. School physical activity policy or procedure:** |  |  |
| Does your school have a written physical activity policy relating to student physical activity across the school environment and curriculum? (this is not the same as a sport policy) | 1. Yes  2. No  3. Don’t know | Yes |
| Does the policy specify the minimum minutes of physical activity students are provided with throughout the school week? | 1. Yes  2. No  3. Don’t know | Yes |
| What are the minimum minutes? | Open | 150 or more |
| Does the policy:  -support the use of a set of written principles to maximize PE quality, active PE time and student engagement in PE lessons?  -encourage the development of yearly personal PA plans for each student;  -mention the school running an Enhanced School sport program focused on building students’ physical activity skills; -mention having supervised and planned activities and equipment provision at recess and lunch;  -mention developing links with community organisations to enhance student’s physical activity opportunities outside of school;  -mention informing parents about physical activity recommendations, physical activity policies, programs or partnerships occurring within or with the school? | 1. Yes  2. No  3. Don’t know | Yes to at least 3 of the 6 |
| **6. Links with community physical activity providers:** |  |  |
| These questions are about community-based physical activity providers. E.g. individual or team sports groups, fitness industry and gyms, sporting or physical activity facilities.  Does your school promote any community physical activity providers to students and families (e.g. in school newsletters) (this does not include any that are used solely for School sport, PE or other physical activities within school time). If so, how many have been promoted? | 1. No  2. Yes 1-4  3. 5-9  4. 10-15  5. >15 | Question did not contribute to criteria. It was asked to assist respondents differentiate promotion from more formal links and provided additional data. |
|  |  |  |
| The next questions are about more formal links between the school and community-based physical activity providers that go beyond promotion. These links involve a connection, partnership or engagement between the school and a provider, where the provider works jointly with the school (e.g. uses the school facilities after hours, runs introductory sessions at the school or for school students, or makes presentations to students at schools, offers transport to the venue or free/low cost introductory offers for school students. You may have a contract or agreement with the provider for such a service.  Does your school have any more formal links with community physical activity providers? | 1. Yes  2. No  3. Don’t know | Yes |
| Please name the provider/s. | Open | Nominate at least 3 providers |
| Is the provider promoted or the link communicated to students and their families? | 1. Yes  2. No  3. Don’t know | Yes |
| If so, how often? | 1. Yes, once term  2. Yes, once semester  3. Yes, once year  4. No  5. Don’t know | Yes, once term |
| *Are there activities offered by this provider that are free (no/low cost) for students?******.* | 1. Yes in an ongoing way  2. Yes limited/short term (e.g. introductory offer)  3. No  4. Don’t know | *Yes, in an ongoing way for at least one link (for desirable)* |
| **7. Communicating physical activity messages to all parents.** |  |  |
| Has your school provided information to parents about physical activity (this does not include advertisement of community physical activity providers, student or school sporting performances, or upcoming school sports events or timetables) | 1. Yes  2. No  3. Don’t know | Yes |
| How often? | 1. Once per term  2. Once per semester  3. Once per year  4. Don’t know | Once per term |
