# Supplementary material for: Scale-up of the Physical Activity 4 Everyone (PA4E1) intervention in secondary schools: 24-month implementation and cost outcomes from a cluster randomised controlled trial
Source: Int J Behav Nutr Phys Act. 2021 Oct 23;18:137. doi: 10.1186/s12966-021-01206-8 (PMC8542325; doi:10.1186/s12966-021-01206-8)
Supplement: Supplementary file 6 — Additional file 6 : Supplementary File 6. Fidelity, reach and per-protocol analyses. [file 12966_2021_1206_MOESM6_ESM.docx]

## Supplementary File 6

### Criteria and data source for fidelity and reach at 0-12 months and 12-24 months

The number of program schools provided or offered (fidelity) each implementation support sub-strategy (n=23) as it was prescribed in the original protocol; and the number taking up each implementation support sub-strategy (reach) was calculated (criteria for meeting each sub-strategy see Supplementary File 6, Table 1 and Table 2). Fidelity and reach scores were calculated for each school as follows, allowing equal weighting of the sub-strategies within each strategy, and equal weighting of each strategy within the total. Schools meeting each sub-strategy were given a score of one, and schools not meeting a score of zero, and the total number of sub-strategies met converted to a percentage for that strategy (e.g. 75% if 3 of 4 sub-strategies met). The mean percentage across strategies composed the school’s total score. The means across all schools were calculated to summarise overall fidelity and reach at 0-12, 12-24 and both 0-12 and 12-24 months. Sub-strategy 3.3 was not included in calculations, as the data was included in sub-strategy 3.4 and 6.1, so were not counted twice.

### Per protocol analyses

#### Methods

Per protocol analyses were undertaken for the primary outcome and one secondary outcome (mean number of practices), based on those schools who received the full implementation support intervention (defined as median reach score or above) against controls, using analyses exact logistic regression and linear regression for the respective outcomes between 0-24 months. As no per protocol analyses were published with the 12-month paper, the paper presents analyses for the 0-12 month period and for the 0-24 month period.

At 12- and 24-months, the program schools with reach scores at or above the median (n=13 at 12-months and 24-months) were compared to all control schools on the primary outcome (four or more practices) and the mean number of practices.

#### Additional Results

The median reach score for the 0-12 month period was 82.1%. At 12-month follow-up, significantly more per-protocol program schools had implemented four of the seven practices (10/13, 76.9%) than control group schools (1/25, 4.0%) (p<0.001). After adjusting for baseline differences, the per-protocol program group was implementing a mean of 3.5 practices more than the control group at 12-months (p<0.001, mean 4.2 (SD 1.2)). These results are consistent with the main intention to treat analyses (see Table 4). Similar analysis were undertaken comparing the 13 schools with reach scores at median or above for the whole 0-24 month period (scores at or above 76.2%). Results were again consistent with the main intention to treat analyses (see Table 4).

### Supplementary File 6, Table 1 – Criteria and data source for fidelity and reach at 0-12 months

|  | | | |
| --- | --- | --- | --- |
| **Implementation Support Strategy** | **Data Source(s)** | **Criteria Fidelity (provided)** | **Criteria Reach (Received)** |
| 1. **Executive and leadership support** | | | |
| 1.1: PA4E1 Partnership agreement signed by school executive. | Administrative data | Not Applicable – the implementation team have no role in this part of the support strategy other than providing an agreement document and contacting the school to prompt review/return. | Each school has a signed partnership agreement by school executive saved in the implementation team project files. |
| 1.2: New or existing school committee formed to oversee program. | Head PE Teacher Survey (via Computer Assisted Telephone interview (CATI) at 12-months) | Not Applicable – the implementation team have no role in this part of the support strategy other than encouraging schools to form a committee during initial program training. | If answered “yes” to “Does your school have a committee that facilitates the implementation of physical activity policies and programs in your school?” |
| 1.3: The School committee is inclusive of in-School Champion and school executive to oversee the program. | Head PE Teacher Survey (via CATI at 12-months) | Not Applicable – the implementation team have no role in this part of the support strategy other than encouraging schools to form a committee during initial program training. | If answered “School executive (e.g. Principal)” to “Does your school committee include:” |
| 1.4: Committee met at least once per term. | PA4E1 termly survey (via program website, PA4E1 Online) | Not Applicable– the implementation team have no role in this part of the support strategy other than encouraging schools to form a committee during initial program training. | If answered “yes” in all four school terms (Term 4 2017, Term 1-3 2018) to “Has your school committee (that facilitates the implementation of physical activity policies and programs in your school) met at least once this term?” |
| **2. Embedded school staff: in-School Champion** | | | |
| 2.1: An existing school PE teacher is allocated the role of in-School Champion to support implementation for full 12-months. | Administrative data | Not Applicable– the implementation team have no role in this part of the support strategy. | Each school has a signed partnership agreement by school executive saved in the implementation team project files that names an in-School Champion to lead the project AND the implementation team do not log within project context monitoring log any periods of time that a school doesn’t have an in-School Champion. |
| 2.2: The position was funded by the NSW Department of Health, half day per week (equivalent to $350AUD a fortnight). | Administrative data | Email contact with schools indicates that funding equivalent to $350AUD a fortnight was offered. | Implementation team financial records of money being transferred to schools. |
| **3. External implementation support** | | | |
| 3.1: Health Promotion Support Officer (ideally a trained PE teacher) appointed to support schools with the program. | Administrative data | Implementation team context monitoring log indicates school was supported for all four school terms by a Support Officer regardless of location (Term 4 2017, Term 1-3 2018). | Same criteria as Fidelity. |
| 3.2: Health Promotion Support Officer was co-located within the relevant local health district. | Administrative data | Implementation team context monitoring log indicates school was supported for all four school terms by a Support Officer located within same local health district as the school (Term 4 2017, Term 1-3 2018). | Same criteria as Fidelity. |
| 3.3: Weekly contact was made with in-School Champion via phone, email and/or face-to-face site visits for 12-months. | Not Applicable | Included at support strategy 6.1 and not here. | Included at support strategy 6.1 and not here. |
| 3.4: Support Officer and in-School Champion have a face-to-face contact at least once a term. | Administrative data | Email archives from implementation team (inclusive of Support Officers) to in-School Champions indicates that schools were invited to a face-to-face launch workshop (Term 4 2017) AND at least three face to face visits across Terms 1-3, 2018. | Attendance logs indicate that both in-School Champion and corresponding Support Officer attended face-to-face PA4E1 launch workshop (Term 4 2017) (or alternatively, Support Officers met with in-School Champions face-to-face in Term 4 2017); AND, Support Officers log at least three face-to-face contacts with each school during the three school terms (Term 1-3 2018). |
| **4. Teacher professional learning** | | | |
| 4.1: In-School Champion training –1-day of face to face training session was hosted by PA4E1 implementation team in Term 1. Accommodation, meals and transport costs were covered by the NSW Department of Health. | Administrative data | Email archives from implementation team (inclusive of Support Officers) to in-School Champions indicates that schools were invited to a face-to-face launch workshop (Term 4 2017) AND schools were offered via email their accommodation and meals whilst attending the workshop, and reimbursement for transport costs. | Attendance logs indicate that both in-School Champion and corresponding Support Officer attended face-to-face PA4E1 launch workshop (Term 4 2017) AND financial records indicate that accommodation, transport and meals were paid for by those who requested it via email. |
| 4.2: Quality PE training for all PE teachers - 6 x 10-minute online training videos followed by knowledge check short quizzes focused on the SAAFE principles were delivered via a password protected program website. | PA4E1 program website AND termly survey (via program website, PA4E1 Online) | Program website (PA4E1 Online) provides access to all PE teachers and in-School Champions at schools to complete online training modules and associated quizzes. | Termly survey via program website (PA4E1 Online) indicates at least 70%* of PE teachers and in-School Champions at schools have completed online training modules and associated quizzes.  *70% used as a cut-off during implementation as benchmark rather than ‘all’ registered users of the program website. |
| 4.3: Enhanced school sport training – in-School Champions and other teachers involved in delivering the program could attend an existing 1 day face-to-face Resistance Training for Teens workshop offered by the NSW Department of Education (School Sport Unit), or equivalent training run by PA4E1 implementation team (not accredited). Course costs to be paid by project for in-School Champion, but not for other teachers. | Administrative data | Email archives from implementation team (inclusive of Support Officers) indicate that all staff who will deliver Resistance Training for Teens were offered access to attend a 1 day face-to-face training for Resistance Training for Teens, and funding was offered to cover the cost of the in-School Champion. | Implementation team attendance monitoring logs (Microsoft OneNote) indicate that at least one staff member at each school has attended a 1-day Resistance Training for Teens workshop, AND, financial records show that the cost of the training was paid for by the project for in-School Champions to attend. |
| 4.4: School physical activity policy training – in-School Champion offered existing online training run by the NSW Department of Education School Sport Unit (Government schools only, n=19) ([43](#_ENREF_43)). | Administrative data AND NSW Department of Education School Sport Unit training completion data. | Email archives from implementation team (inclusive of Support Officers) indicate that all in-School Champions (Government schools only) were invited to complete NSW Department of Education School Sport Unit School physical activity policy training within first four terms of the program (Term 4 2017, Term 1-3 2018). | NSW Department of Education School Sport Unit completion records indicate that in-School Champions (Government Schools only) have completed the NSW Department of Education School Sport Unit School physical activity policy training within first four terms of the program (Term 4 2017, Term 1-3 2018). |
| **5. Resources** | | | |
| 5.1: Printed posters outlining Quality PE principles (SAAFE Principles ([44](#_ENREF_44))) to be displayed in PE department delivered to in-School Champions. | Administrative data | Implementation team project records via Microsoft Excel indicate that schools were sent (via mail or Support Officer) a set of printed SAAFE posters. | Same criteria as Fidelity. |
| 5.2: A $100AUD physical activity equipment voucher was provided to support the delivery of recess and lunchtime physical activity. | Administrative data | Email archives from implementation team (inclusive of Support Officers) indicate that in-School Champions were offered a $100AUD voucher for a physical activity equipment supplier. | Email archives indicate that in-School Champions at schools were sent a $100AUD voucher for a physical activity equipment supplier. |
| 5.3: Equipment provided to support the delivery of recess and lunchtime physical activity enhanced schools sport program (5 Gymsticks/school) | Administrative data | Email archives from implementation team (inclusive of Support Officers) indicate that in-School Champions* were offered a set of five gymsticks.  *Schools were only offered the five gymsticks if they provided evidence to the implementation team of programing an enhanced school sport program (Resistance Training for Teens). | Implementation team project records via Microsoft Excel indicate that schools were sent (via mail or Support Officer) a set of five gymsticks. |
| 5.4: Electronic resources housed on the program website (PA4E1 online) included:   - - overview of program presentation (Microsoft PowerPoint presentation),   - project milestones to be achieved each term (over 4 terms),   - online quality PE training (SAAFE Principle videos (6 videos - one overview and one per Principle) and worksheet, peer observation materials),   - student personal physical activity plan templates,   - recess and lunch resources,   - policy templates,   - examples of community physical activity providers,   - tips and frequently asked questions | PA4E1 program website (PA4E1 Online) | Email archives from PA4E1 Implementation Team indicate that schools were offered PA4E1 Online access. | PA4E1 program website content management system (Umbraco) indicates that at least the in-School Champion and at one PE teacher per school register in PA4E1 Online. |
| **6. Provision of prompts and reminders** | | | |
| 6.1: Weekly emails or phone calls were made by the Support Officer to in-School Champions to encourage implementation. | Administrative data | Implementation team (Support Officers) log at 8* contacts term via email, phone or face-to-face with each school across each of three** school terms (Term 1-3 2018).  *8 contacts per 10 week term was used as an operational definition of weekly contact, as a reasonable reflection of weekly contact in a 10 week term, allowing for teacher absences etc.  **three rather than four school terms used as data on contacts was not collected in Term 4 2017 | Same criteria as Fidelity. |
| 6.2: Automated messages were sent each term via the program website to in-School Champions to prompt completion of teacher professional learning and online termly performance monitoring and feedback surveys. | PA4E1 program website (PA4E1 Online) AND Administrative data | PA4E1 program website content management system (Umbraco) indicates that automated messages were set up. | PA4E1 program website content management system (Umbraco) indicates that automated messages were set up AND a Support Officer initiated logic check with a sub-sample of in-School Champions suggests that they are receiving these emails. |
| **7. Implementation performance monitoring and feedback** | | | |
| 7.1 In-School Champion completes all termly surveys via the program website (PA4E1 Online). | PA4E1 program website (PA4E1 Online) | PA4E1 content management system (Umbraco) indicates that termly surveys were available to be completed by in-School Champions each term. | Program website (PA4E1 Online) indicates that termly surveys were completed by in-School Champions each of the four school terms (Term 4 2017, Term 1-3 2018). |
| 7.2 A feedback report is automatically generated and sent to in-School Champions via email | PA4E1 program website (PA4E1 Online) | Program website (PA4E1 Online) indicates that termly survey feedback reports were available for in-School Champions for each of the school terms they completed a termly survey for between Term 4 2017 and Term 3 2018) | Same criteria as Fidelity. |
| 7.3 A feedback report is automatically generated and sent to school Principals via email | PA4E1 program website (PA4E1 Online) | Program website (PA4E1 Online) indicates that termly survey feedback reports were available for school Principals for each of the school terms they completed a termly survey for between Term 4 2017 and Term 3 2018) | For Principals verified in PA4E1, the Program website (PA4E1 Online) indicates that termly survey feedback reports were available for school Principals for each of the school terms they completed a termly survey for between Term 4 2017 and Term 3 2018) |

### Supplementary File 6, Table 2 – Criteria and data source for fidelity and reach at 12-24 months

|  | | | |
| --- | --- | --- | --- |
| **Implementation Support Strategy** | **Data Source(s)** | **Criteria Fidelity (provided)** | **Criteria Reach (Received)** |
| 1. **Executive and leadership support** | | | |
| 1.1: PA4E1 Partnership agreement signed by school executive. | Administrative data | Not Applicable – the implementation team have no role in this part of the support strategy other than providing an agreement document and contacting the school to prompt review/return. | Each school has a signed partnership agreement by school executive saved in the implementation team project files. |
| 1.2: New or existing school committee formed to oversee program. | Head PE Teacher Survey (via Computer Assisted Telephone interview (CATI) at 24-months) | Not Applicable – the implementation team have no role in this part of the support strategy other than encouraging schools to form a committee during initial program training. | If answered “yes” to “Does your school have a committee that facilitates the implementation of physical activity policies and programs in your school?” |
| 1.3: The School committee is inclusive of in-School Champion and school executive to oversee the program. | Head PE Teacher Survey (via CATI at 24-months) | Not Applicable – the implementation team have no role in this part of the support strategy other than encouraging schools to form a committee during initial program training. | If answered “School executive (e.g. Principal)” to “Does your school committee include:” |
| 1.4: Committee met at least once per term. | PA4E1 termly survey (via program website, PA4E1 Online) | Not Applicable– the implementation team have no role in this part of the support strategy other than encouraging schools to form a committee during initial program training. | If answered “yes” in all four school terms (Term 4 2018, Term 1-3 2019) to “Has your school committee (that facilitates the implementation of physical activity policies and programs in your school) met at least once this term?” |
| **2. Embedded school staff: in-School Champion** | | | |
| 2.1: An existing school PE teacher is allocated the role of in-School Champion to support implementation for full 12-months (term 4 2018 and terms 1-3 2019). | Administrative data | Not Applicable– the implementation team have no role in this part of the support strategy. | Each school has a signed partnership agreement by school executive saved in the implementation team project files that names an in-School Champion to lead the project AND the implementation team do not log within project context monitoring log any periods of time that a school doesn’t have an in-School Champion. |
| 2.2: The position was funded by the NSW Department of Health, half day per week (equivalent to $350AUD a fortnight). | Administrative data | Email contact with schools indicates that funding equivalent to $350AUD a fortnight was offered. | Implementation team financial records of money being transferred to schools. |
| **3. External implementation support** | | | |
| 3.1: Health Promotion Support Officer (ideally a trained PE teacher) appointed to support schools with the program. | Administrative data | Implementation team context monitoring log indicates school was supported for all four school terms by a Support Officer regardless of location (Term 4 2018, Term 1-3 2019). | Same criteria as Fidelity. |
| 3.2: Health Promotion Support Officer was co-located within the relevant local health district. | Administrative data | Implementation team context monitoring log indicates school was supported for all four school terms by a Support Officer located within same local health district as the school (Term 4 2018, Term 1-3 2019). | Same criteria as Fidelity. |
| 3.3: Contact was made with in-School Champion via phone, email and/or face-to-face site visits according to the following high dose/low dose protocol matched to school practice uptake in the preceding term (e.g. term 5 informs term 6)  a. Schools implementing *four or less practices* received 2 face-to-face contact points, as well as 6 emails or phonecalls in the term.  b. Schools implementing *five or more practices* 1 face-to-face contact point and seven emails or phone calls in the term. | Not Applicable | Included at support strategy 6.1 and not here. | Included at support strategy 6.1 and not here. |
| 3.4: Support Officer and in-School Champion have a face-to-face site visits according to the following high dose/low dose protocol matched to school practice uptake in the preceding term (e.g. term 5 informs term 6)  a. Schools implementing *four or less practices* received 2 face-to-face contact points  b. Schools implementing *five or more practices* 1 face-to-face contact point | Administrative data | Email archives from implementation team (inclusive of Support Officers) to in-School Champions indicates that schools were invited to a face-to-face workshop (Term 4 2018) AND at least three face to face visits across Terms 1-3, 2019 (but up to six, see criteria for high dose and low dose support). | Attendance logs indicate that both in-School Champion and corresponding Support Officer attended face-to-face PA4E1 workshop (Term 4 2018) (or alternatively, Support Officers met with in-School Champions face-to-face in Term 4 2018); AND, Support Officers log at least three face-to-face contacts with each school during the three school terms: Term 1-3 2019 (but up to six, see criteria for high dose and low dose support). |
| **4. Teacher professional learning** | | | |
| 4.1: In-School Champion training –1-day of face to face training session was hosted by PA4E1 implementation team in Term 4, 2018. Accommodation, meals and transport costs were covered by the NSW Department of Health. | Administrative data | Email archives from implementation team (inclusive of Support Officers) to in-School Champions indicates that schools were invited to a face-to-face launch workshop (Term 4 2018) AND schools were offered via email their accommodation and meals whilst attending the workshop, and reimbursement for transport costs. | Attendance logs indicate that both in-School Champion and corresponding Support Officer attended face-to-face PA4E1 launch workshop (Term 4 2018) AND financial records indicate that accommodation, transport and meals were paid for by those who requested it via email. |
| 4.2: Quality PE training for all PE teachers - 6 x 10-minute online training videos followed by knowledge check short quizzes focused on the SAAFE principles were delivered via a password protected program website. | PA4E1 program website AND termly survey (via program website, PA4E1 Online) | Program website (PA4E1 Online) provides access to all PE teachers and in-School Champions at schools to complete online training modules and associated quizzes. | Termly survey via program website (PA4E1 Online) indicates at least 70%* of PE teachers and in-School Champions at schools have completed online training modules and associated quizzes.  *70% used as a cut-off during implementation as benchmark rather than ‘all’ registered users of the program website. |
| 4.3: Enhanced school sport training* – in-School Champions and other teachers involved in delivering the program could attend an existing 1 day face-to-face Resistance Training for Teens workshop offered by the NSW Department of Education (School Sport Unit), or equivalent training run by PA4E1 implementation team (not accredited). Course costs to be paid by project for in-School Champion, but not for other teachers.  *Only offered in the first 12-months | Administrative data | Email archives from implementation team (inclusive of Support Officers) indicate that all staff who will deliver Resistance Training for Teens were offered access to attend a 1 day face-to-face training for Resistance Training for Teens, and funding was offered to cover the cost of the in-School Champion. | Implementation team attendance monitoring logs (Microsoft OneNote) indicate that at least one staff member at each school has attended a 1-day Resistance Training for Teens workshop, AND, financial records show that the cost of the training was paid for by the project for in-School Champions to attend. |
| 4.4: School physical activity policy training* – in-School Champion offered existing online training run by the NSW Department of Education School Sport Unit (Government schools only, n=19) ([43](#_ENREF_43)).  *Only offered in the first 12-months | Administrative data AND NSW Department of Education School Sport Unit training completion data. | Email archives from implementation team (inclusive of Support Officers) indicate that all in-School Champions (Government schools only) were invited to complete NSW Department of Education School Sport Unit School physical activity policy training within first four terms of the program (Term 4 2017, Term 1-3 2018). | NSW Department of Education School Sport Unit completion records indicate that in-School Champions (Government Schools only) have completed the NSW Department of Education School Sport Unit School physical activity policy training within first four terms of the program (Term 4 2017, Term 1-3 2018). |
| **5. Resources** | | | |
| 5.1: Printed posters outlining Quality PE principles (SAAFE Principles ([44](#_ENREF_44))) to be displayed in PE department delivered to in-School Champions.  *Only offered in the first 12-months | Administrative data | Implementation team project records via Microsoft Excel indicate that schools were sent (via mail or Support Officer) a set of printed SAAFE posters. | Same criteria as Fidelity. |
| 5.2: A $100AUD physical activity equipment voucher was provided to support the delivery of recess and lunchtime physical activity.  *Only offered in the first 12-months | Administrative data | Email archives from implementation team (inclusive of Support Officers) indicate that in-School Champions were offered a $100AUD voucher for a physical activity equipment supplier. | Email archives indicate that in-School Champions at schools were sent a $100AUD voucher for a physical activity equipment supplier. |
| 5.3: Equipment provided to support the delivery of recess and lunchtime physical activity enhanced schools sport program (5 Gymsticks/school)  *Only offered in the first 12-months | Administrative data | Email archives from implementation team (inclusive of Support Officers) indicate that in-School Champions* were offered a set of five gymsticks.  *Schools were only offered the five gymsticks if they provided evidence to the implementation team of programing an enhanced school sport program (Resistance Training for Teens). | Implementation team project records via Microsoft Excel indicate that schools were sent (via mail or Support Officer) a set of five gymsticks. |
| 5.4: Electronic resources housed on the program website (PA4E1 online) included:   - - overview of program presentation (Microsoft PowerPoint presentation),   - project milestones to be achieved each term (over 4 terms),   - online quality PE training (SAAFE Principle videos (6 videos - one overview and one per Principle) and worksheet, peer observation materials),   - student personal physical activity plan templates,   - recess and lunch resources,   - policy templates,   - examples of community physical activity providers,   - tips and frequently asked questions | PA4E1 program website (PA4E1 Online) | Email archives from PA4E1 Implementation Team indicate that schools were offered PA4E1 Online access. | PA4E1 program website content management system (Umbraco) indicates that at least the in-School Champion and at one PE teacher per school register in PA4E1 Online. |
| **6. Provision of prompts and reminders** | | | |
| 6.1: Contact was made by the Support Officer to in-School Champions via phone, email and/or face-to-face site visits according to the following high dose/low dose protocol matched to school practice uptake in the preceding term (e.g. term 5 informs term 6)  a. Schools implementing *four or less practices* received 2 face-to-face contact points, as well as 6 emails or phonecalls in the term.  b. Schools implementing *five or more practices* 1 face-to-face contact point and seven emails or phone calls in the term. | Administrative data | Implementation team (Support Officers) log contacts via email, phone or face-to-face with each school across each of four school terms (Term 4 2018, terms 1-3 2019) according to the high dose/low dose protocol. | Same criteria as Fidelity. |
| 6.2: Automated messages were sent each term via the program website to in-School Champions to prompt completion of teacher professional learning and online termly performance monitoring and feedback surveys. | PA4E1 program website (PA4E1 Online) AND Administrative data | PA4E1 program website content management system (Umbraco) indicates that automated messages were set up. | PA4E1 program website content management system (Umbraco) indicates that automated messages were set up AND a Support Officer initiated logic check with a sub-sample of in-School Champions suggests that they are receiving these emails. |
| **7. Implementation performance monitoring and feedback** | | | |
| 7.1 In-School Champion completes all termly surveys via the program website (PA4E1 Online) (term 4 2018, terms 1-3 2019). | PA4E1 program website (PA4E1 Online) | Termly surveys were available to be completed by in-School Champions for each term. | Program website (PA4E1 Online) indicates that termly surveys were completed by in-School Champions each of the four school terms (Term 4 2018, Term 1-3 2019). |
| 7.2 A feedback report is automatically generated and sent to in-School Champions via email | PA4E1 program website (PA4E1 Online) | Program website (PA4E1 Online) indicates that termly survey feedback reports were available for in-School Champions for each of the school terms they completed a termly survey for between Term 4 2018 and Term 3 2019) | Same criteria as Fidelity. |
| 7.3 A feedback report is automatically generated and sent to school Principals via email | PA4E1 program website (PA4E1 Online) | Program website (PA4E1 Online) indicates that termly survey feedback reports were available for school Principals for each of the school terms they completed a termly survey for between Term 4 2018 and Term 3 2019) | For Principals verified in PA4E1, the Program website (PA4E1 Online) indicates that termly survey feedback reports were available for school Principals for each of the school terms they completed a termly survey for between Term 4 2018 and Term 3 2019) |
