# Supplementary material for: Scale-up of the Physical Activity 4 Everyone (PA4E1) intervention in secondary schools: 24-month implementation and cost outcomes from a cluster randomised controlled trial
Source: Int J Behav Nutr Phys Act. 2021 Oct 23;18:137. doi: 10.1186/s12966-021-01206-8 (PMC8542325; doi:10.1186/s12966-021-01206-8)
Supplement: Supplementary file 7 — Additional file 7 : Supplementary File 7. Economic evaluation. [file 12966_2021_1206_MOESM7_ESM.docx]

## Supplementary File 7

### Economic Evaluation Methods

The Industrial Relations Commission of NSW 2017 Award data were used to value labour costs for teaching personnel and External Support Officer employed by the NSW Department of Health (1). With respect to control schools, it was assumed that no additional costs were incurred in implementing their usual physical activity practices.

Resource use associated with the intervention was prospectively identified, measured and valued over the course of the trial. Resource use data were collected over the intervention period via a bespoke cost capture tool developed in MS Excel (2013). The tool allowed the input of resource use data from team members involved in implementing the intervention for the following cost categories: labour (health service (including research team) and non-health service (such as school personnel), materials, joint costs and miscellaneous costs. Management logs routinely used by the external Support Officers were also completed weekly to capture labour, school contacts and resource use. The resource use capture tool has previously been used to evaluate intervention costs and evaluate cost-effectiveness within the PA4E1 efficacy trial (2).

### Economic Analysis Methods

Cost and cost-effectiveness analysis were undertaken from a public finance perspective and all analyses were carried out using Microsoft Excel 2013. The analysis was conducted on an intention to treat basis, with the total intervention cost being calculated across all program schools (n=24) and intervention strategies. To present the intervention mean cost per school, the total intervention cost over the 24-month period was divided by the number of program schools (n=24). The incremental cost-effectiveness ratio (ICER) was calculated for the primary outcome representing the cost per percent change in the proportion of schools implementing at least four of the seven practices. The mean intervention cost per school was divided by difference in change in proportion of schools implementing at least four of the seven physical activity practices (primary trial outcome). Sensitivity and uncertainty analysis were used to account for uncertainty due to sampling variation, nonparametric bootstrapping analysis with 1000 iterations. The bootstrapped ICERs were graphically mapped on a cost-effectiveness plane (Supplementary File 7, Figure 1).


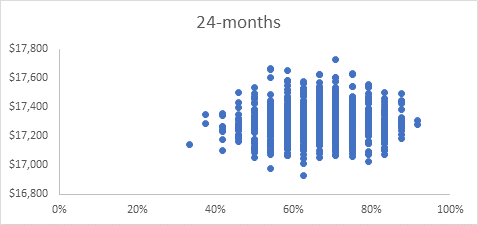


**Supplementary File 7, Figure 1.** ICER pairs mapped onto a cost-effectiveness plane, at 24-months.

**Supplementary File 5, Table 1.** PA4E1 Implementation support strategy cost per year and mean cost per school

| ***Implementation support strategies (n=7) (implemented over 8 school terms)*** | **Description of costs** | **Total cost on implementation support intervention** | | | **Mean cost per school** | |
| --- | --- | --- | --- | --- | --- | --- |
|  |  | **Cost**  **0-12 months** | **Cost**  **12-24 months** | **Total costs over 24 months** | **Mean cost per school over 12 months (n=24)** | **Mean cost per school over 24 months (n=24)** |
| 1. Executive and leadership support | Partnership agreement signed, School committee established. School executive membership represented on committee. Cost includes the opportunity cost of school staff time associated with committee meeting attendance, reviewing policy and performance feedback reports. | $1,286 | $1,318 | $2,604 | $53.60 | $108.50 |
| 2. Embedded school staff: in-School Champion | Teacher relief funding provided to the school for ½ day per week for 2 years | $168,000 | $168,000 | $336,000 | $7,000.00 | $14,000.00 |
| 3. External implementation support^#^ | Cost to provide weekly face-to-face, phone or email support to schools in the program group. The costs cover actual Support Officer salary (time), travel and accommodation costs for external Support Officer. | $7,909 | $9,665 | $17,574 | $329.53 | $723.50 |
| 4. Teacher professional learning | Two day joint school professional development training held centrally once per year. Cost includes the opportunity cost of school staff time (teacher relief), travel and meal expenses and venue hire | $4,750 | $9,369 | $14,119 | $197.92 | $588.29 |
| 5. Resources | Provision of resources to school including gymsticks for resistance training, equipment vouchers for recess and lunchtime equipment and posters printed for PE staffrooms. | $15,214 | $3,391 | $18,605 | $633.92 | $775.21 |
| 6. Provision of prompts and reminders | Costed within external implementation Support Officer time above and automated within the PA4E1 online portal | $0 | $0 | $0 | $0 | $0 |
| 7. Implementation performance monitoring and feedback | Report delivered 1 x per term (n=8) to Principal and head PE teacher. Costs are estimated for time taken for the school principal and school champion to review termly report. | $195 | $195 | $390 | $8.12 | $16.25 |
| Additional centralised resources | Cost to develop and maintain the PA4E1 online portal used by all schools in the program group to automate the delivery of the implementation strategies and house resources electronically. | $24,700 | $1,120 | $25,820 | $1,029.16 | $1,075 |
| **Total costs** |  | **$222,054** | **$193,058** | **$415,112** | **$9,252.25** | **$17,296.33** |

## References

1. New South Wales Industrial Relations Commission (Industrial Gazette) (2017). Accessed 31/03/2021. Available from: <http://www.lawlink.nsw.gov.au/irc/ircgazette.nsf/(PublicationsByTitle)/FBA6661A7F84272BCA2581160011216A?OpenDocument>.

2. Sutherland R, Reeves P, Campbell E, Lubans DR, Morgan PJ, Nathan N, et al. Cost effectiveness of a multi-component school-based physical activity intervention targeting adolescents: the 'Physical Activity 4 Everyone' cluster randomized trial. The international journal of behavioral nutrition and physical activity. 2016;13:94.
