# Supplementary material for: Scale-up of the Physical Activity 4 Everyone (PA4E1) intervention in secondary schools: 24-month implementation and cost outcomes from a cluster randomised controlled trial
Source: Int J Behav Nutr Phys Act. 2021 Oct 23;18:137. doi: 10.1186/s12966-021-01206-8 (PMC8542325; doi:10.1186/s12966-021-01206-8)
Supplement: Supplementary file 8 — Additional file 8 : Supplementary File 8. Practice implementation for each practice. [file 12966_2021_1206_MOESM8_ESM.docx]

## Supplementary File 8

Table 1 below shows the number of program group schools meeting practices at neither or both of the 12- and 24-month follow-ups. The number of program schools meeting the four or more practices criteria: at neither 12- or 24-months (4/23, 17.4%); at 12-months only (3/23, 13%), at 24-months only (4/23, 17.4%), and at both 12- and 24-months (12/23, 52.2%). The figures for control schools were at neither 12- nor 24-months 24/25, 96%); at both 12- and 24-months 1/25, 4%.

**Supplementary File 8, Table 1:** Number of program group schools implementing practices at neither 12- nor 24-month time points, 12-month only, 24-month only, and both 12- and 24-months (N=23)

|  | **Program group schools (N=23) implementation of each practice** | | | |
| --- | --- | --- | --- | --- |
|  | **Neither 12- or 24-months**  **n (%)** | **12-month only**  **n (%)** | **24-month only**  **n (%)** | **Both 12- and 24-months n (%)** |
| 4 or more PA practices | 4 (17.4) | 3 (13.0) | 4 (17.4) | 12 (52.2%) |
| **Individual practices** |  |  |  |  |
| 1. Quality PE lessons | 6 (26.1) | 3 (13.0) | 4 (17.4) | 10 (43.5%) |
| 2. Student PA plans: | 1 (4.3) | 3 (13.0) | 2 (8.7) | 17 (74.9%) |
| 3. Enhanced school sport program: | 3 (13.0) | 4 (17.4) | 1 (4.3) | 15 (65.2%) |
| 4. Recess/ lunchtime physical activity: | 6 (26.1) | 3 (13.0) | 6 (26.1) | 8 (34.8%) |
| 5. School PA policy or procedure: | 10 | 4 (17.4) | 7 (30.4) | 2 (8.7%) |
| 6. Links with community PA providers | 21 (91.3) | 0 (0.0) | 2 (8.7) | 0 (0.0%) |
| 7. Communicating PA messages to all parents | 2 (8.7) | 1 (4.3) | 2 (8.7) | 18 (78%) |
